# Corticosteroid insensitivity in obese asthma: potential mechanisms and therapeutic perspectives

**DOI:** 10.3389/falgy.2025.1719900

**Published:** 2025-11-25

**Authors:** Masako To, Yasuo To

**Affiliations:** 1 Department of Laboratory Medicine, Dokkyo Medical University, Saitama Medical Center, Koshigaya, Saitama, Japan; 2 Department of Pulmonary Medicine, International University of Health and Welfare Narita Hospital, Narita, Chiba, Japan

**Keywords:** adult asthma, drug-repositioning, obesity, oxidative stress, steroid insensitivity

## Abstract

Asthma is a heterogeneous condition influenced by multiple clinical and biological factors, and obesity has emerged as a major modifier that worsens symptoms and increases the risk of exacerbations. This review aimed to examine the mechanisms by which obesity contributes to reduced responsiveness to corticosteroids, which remain the cornerstone of guideline-based asthma management. We reviewed evidence from clinical and experimental studies describing how adipose tissue dysfunction, chronic low-grade inflammation, oxidative stress, and systemic comorbidities alter glucocorticoid receptor signalling and downstream pathways. Particular attention was given to immune mechanisms such as neutrophilic inflammation and interleukin-17 signalling, as well as metabolic disturbances including hyperleptinaemia and vitamin D deficiency. We also considered the role of lifestyle factors, such as physical inactivity and dietary patterns, in sustaining corticosteroid insensitivity. Based on these insights, we evaluated both established and emerging therapeutic strategies, including weight loss, structured exercise, dietary modification, and drug repurposing with agents such as metformin, low-dose theophylline, and glucagon-like peptide-1 receptor agonists. A comprehensive synthesis of these findings highlights the need for integrated lifestyle and pharmacological interventions, and provides a framework for the development of targeted treatments to improve outcomes in patients with obesity-associated, corticosteroid-insensitive asthma.

## 1 Introduction

Asthma is a heterogeneous disease characterised by diverse clinical manifestations, inflammatory profiles, and responses to treatment. Among the factors influencing its pathophysiology, obesity has been recognised as a significant contributor to adverse outcomes. Patients with asthma and obesity are at higher risk of acute exacerbations, experience more severe symptoms, and are more likely to require ventilatory support (1–5). Although the epidemiological association between obesity and poor asthma outcomes is well established, the underlying mechanisms remain only partly understood.

Currently, at least two distinct phenotypes have been identified among patients with asthma and obesity (6, 7). The first is a late-onset, non–type 2 phenotype, which is more prevalent in women and is characterised by pronounced oxidative stress. In this phenotype, adipose tissue–derived cytokines and oxidative stress contribute to airway inflammation. Obesity-related alterations in autonomic nervous system function, particularly increased parasympathetic tone, also promote airway narrowing (7). Weight loss in these patients can lead to substantial improvement in asthma control (8). Because the pathophysiological changes driven by obesity appear to directly induce asthma in this group, it has been described as “asthma consequent to obesity.” The second phenotype is early-onset, with elevated immunoglobulin E (IgE) levels and prominent type 2 inflammation (7). This phenotype is interpreted as typical allergic asthma whose disease course is modified by the presence of obesity, and is therefore termed “asthma complicated by obesity.” Although obesity has been reported to amplify eosinophilic inflammation, the precise mechanisms underlying asthma worsening in this phenotype remain incompletely understood (9).

A particularly concerning feature of obesity-associated asthma is the reduced responsiveness to inhaled corticosteroids (ICS), which are a cornerstone of guideline-based asthma management. Reduced corticosteroid responsiveness leads to more frequent exacerbations, often necessitating the use of systemic corticosteroids. The use of systemic corticosteroids can, in turn, contribute to further weight gain and metabolic dysfunction, thereby reinforcing a vicious cycle of poor asthma control and progressive obesity. A clearer mechanistic understanding of corticosteroid resistance in patients with asthma and obesity is crucial for breaking this cycle and developing more effective therapeutic strategies.

In this review, we examine the mechanisms by which obesity may reduce corticosteroid sensitivity in asthma. We first outline obesity-related changes in adipose tissue biology and their potential effects on corticosteroid sensitivity. We then discuss cellular, molecular, and systemic pathways proposed to contribute to corticosteroid insensitivity in patients with asthma and obesity. Finally, we highlight emerging therapeutic approaches aimed at restoring corticosteroid sensitivity and improving clinical outcomes in patients with asthma associated with obesity.

## 2 Corticosteroid resistance in obese asthma: clinical data

Reduced responsiveness to corticosteroid therapy in patients with asthma and obesity has been demonstrated in multiple clinical studies. In a clinical trial involving 3,073 adults with moderate asthma, the response to inhaled beclomethasone decreased with increasing body mass index (BMI), whereas the response to montelukast was observed irrespective of BMI (10). Similarly, a study of 72 patients with mild to moderate persistent asthma reported that overweight individuals exhibited attenuated symptom improvement and reduced fractional exhaled nitric oxide responses to inhaled budesonide compared with normal-weight patients (11). Another study enrolled 1,242 corticosteroid-naïve patients with moderate asthma and evaluated asthma control following treatment with either fluticasone alone or a combination of fluticasone and salmeterol. While the combination therapy was effective overall, obese patients were significantly less likely than non-obese patients to achieve adequate asthma control with either treatment regimen (12). To et al. further assessed corticosteroid sensitivity using peripheral blood mononuclear cells (PBMCs) from patients with asthma and demonstrated that individuals with excessive body fat exhibited significantly reduced corticosteroid sensitivity compared with controls (13). These clinical findings highlight consistent evidence that obesity is linked to impaired corticosteroid responsiveness in asthma, providing a strong foundation for investigating the underlying mechanisms and developing targeted interventions.

## 3 Overview of factors linking pathogenic changes in obese adipose tissue and corticosteroid insensitivity

Adipose tissue plays a fundamental role in maintaining energy homeostasis. Under conditions of energy surplus, adipocytes store excess nutrients as triglycerides, whereas during fasting or starvation, they release fatty acids to meet systemic energy demands. Once regarded as a passive energy reservoir, adipose tissue is now recognised as an active endocrine and immunological system that influences distant organs through the secretion of adipokines, cytokines, and metabolic mediators.

As obesity develops, adipocytes undergo marked hypertrophy due to triglyceride accumulation. This expansion increases lipid droplet size disproportionately relative to cytoplasmic volume, placing mechanical stress on the plasma membrane and straining intracellular organelles such as mitochondria and the endoplasmic reticulum (14–16). These biomechanical and metabolic stresses induce local hypoxia and endoplasmic reticulum stress (17), which alter adipokine production and create an inflammatory microenvironment (18, 19). This inflammatory state is further intensified by a phenotypic shift of resident macrophages from anti-inflammatory M2 to pro-inflammatory M1 phenotypes (20, 21). M1 macrophages secrete cytokines such as interleukin (IL)-1β, IL-6, and tumour necrosis factor-alpha (TNF-α), thereby amplifying local inflammation. The inflammation within adipose tissue exerts systemic effects that contribute to the development of obesity-related complications (22).

In addition to promoting inflammation, adipocyte dysfunction in obesity induces pronounced oxidative stress (23). This arises through several interrelated mechanisms within adipose tissue. Elevated nicotinamide adenine dinucleotide phosphate (NADPH) oxidase activity enhances the production of reactive oxygen species (ROS) (23). Excessive mitochondrial fatty acid oxidation further contributes to ROS accumulation. At the same time, the expression of key antioxidant enzymes—such as superoxide dismutase and catalase—is reduced (23), thereby diminishing the cell's capacity to counteract oxidative damage. These processes establish a pro-oxidant environment that disrupts adipocyte homeostasis. Importantly, increased oxidative stress is observed in individuals with obesity even in the absence of external factors such as cigarette smoking, affecting both adipose tissue and the systemic circulation (23–26).

Several of these alterations in the adipose tissue environment are thought to impair corticosteroid sensitivity. For example, increased oxidative stress and elevated pro-inflammatory cytokines can diminish glucocorticoid receptor (GR) function. Obesity-related oxidative stress can also suppress key regulatory molecules of corticosteroid sensitivity including phosphoinositide 3-kinase (PI3K) and histone deacetylase 2 (HDAC2) (27). Although direct evidence linking obesity-related factors to regulatory molecules of corticosteroid sensitivity remains limited, obesity-induced changes provide a plausible mechanistic link between adipose tissue dysfunction and corticosteroid resistance in asthma.

## 4 Molecular and cellular mechanisms of corticosteroid insensitivity in obese asthma

### 4.1 Glucocorticoid receptor alterations

GRs, the primary binding sites for corticosteroids, exist in multiple isoforms with distinct functions. Among these, GRα is the transcriptionally active form and mediates the classical anti-inflammatory actions of corticosteroids. In contrast, GRβ lacks transcriptional activity and acts as a dominant negative inhibitor of GRα (28). An increased GRβ/GRα ratio has been implicated in the development of corticosteroid insensitivity, and obesity has been reported to be associated with an increased GRβ/GRα ratio (29). One study demonstrated lower GRα expression in obese patients with asthma compared with their non-obese counterparts (30), suggesting a potential link between GR isoform imbalance and reduced corticosteroid responsiveness in this population.

### 4.2 Modification of intracellular signalling pathways

#### 4.2.1 Oxidative stress–related modification of phosphoinositide 3-kinase–Akt and mitogen-activated protein kinase signalling

The PI3K signalling pathway has been widely implicated in the pathogenesis of corticosteroid insensitivity (31–34). Oxidative stress is a key upstream regulator of this pathway. Increased oxidative stress disrupts cell membrane integrity, subsequently activating the PI3K–AKT pathway (32, 35). Once activated, this pathway phosphorylates (HDAC2, inhibiting its enzymatic activity (36). When the glucocorticoid–GR complex translocates into the nucleus, HDAC2 is recruited to suppress pro-inflammatory transcription factors such as NF-κB, thereby attenuating inflammation (37). However, when HDAC2 activity is reduced, GR deacetylation is impaired, diminishing its capacity to repress pro-inflammatory gene transcription. In parallel, oxidative stress activates the mitogen-activated protein kinase (MAPK) pathway. Activation of MAPKs, particularly p38α, p38γ, and JNK1, results in phosphorylation of GRα at sites such as Ser226, which interferes with nuclear translocation and reduces transcriptional repression (35). This mechanism further contributes to corticosteroid insensitivity.

Clinical studies have shown that patients with asthma and obesity exhibit higher systemic oxidative stress compared with non-obese patients (13, 24, 25, 38–40). Notably, in obese patients with asthma, oxidative stress levels correlate with acute exacerbation frequency, whereas no such association has been observed in non-obese patients (39). Moreover, elevated oxidative stress has been associated with reduced corticosteroid sensitivity in obese patients with asthma (13). Although direct evidence implicating obesity-induced PI3K or MAPK activation in corticosteroid insensitivity is not yet available, existing findings strongly suggest that these pathways play a contributory role.

#### 4.2.2 Adenosine monophosphate–activated protein kinase–sirtuin 1 axis

Adenosine monophosphate-activated protein kinase (AMPK) is a ubiquitously expressed serine/threonine kinase in eukaryotes that functions as a key intracellular energy sensor (41). It has also been implicated in oxidative stress tolerance and longevity (42, 43). Sirtuin 1 (SIRT1) is a oxidized form of nicotinamide adenine dinucleotide (NAD^+^)-dependent deacetylase that regulates diverse cellular processes, including metabolism, inflammation, and ageing. AMPK and SIRT1 interact bidirectionally. Activation of AMPK increases the intracellular NAD^+^/reduced form of nicotinamide adenine dinucleotide (NADH) ratio, thereby activating SIRT1 (44). Conversely, SIRT1 activates AMPK by deacetylating lysine residues on an upstream kinase in the AMPK pathway (45).

In obesity, AMPK activity is reduced in adipose tissue (46) due to enhanced inflammation and oxidative stress in hypertrophied adipose tissue. This phenomenon extends to multiple organ systems: studies comparing AMPK activity in PBMCs between obese and non-obese individuals demonstrated lower activity in the obese group (47, 48). Similar reductions in SIRT1 expression have been observed in adipose tissue and PBMCs of obese individuals due to enhanced inflammation and oxidative stress in hypertrophied adipose tissue (49–51). Importantly, dietary weight loss has been shown to restore SIRT1 levels (52), indicating that obesity modulates its expression.

Both AMPK and SIRT1 have been implicated in corticosteroid insensitivity. In patients with chronic obstructive pulmonary disease (COPD), reduced SIRT1 expression in immune cells correlates with diminished corticosteroid sensitivity (53). Furthermore, Mitani et al. reported that PBMCs from patients with COPD exhibited steroid insensitivity, which was reversed upon exposure to an AMPK activator (54), supporting a mechanistic link between AMPK and corticosteroid sensitivity. Collectively, these findings suggest that reduced AMPK and SIRT1 activity in patients with asthma and obesity may contribute to impaired corticosteroid responsiveness.

### 4.3 Potential roles of inflammatory cells and cytokines in corticosteroid insensitivity

#### 4.3.1 Neutrophilic inflammation

In adults with late-onset obesity-related asthma, non–type 2 inflammation is frequently predominant. Sputum analyses have shown increased neutrophil counts in obese patients with asthma compared with non-obese patients (55, 56). Adipose tissue-derived adipocytokines, including IL-6, plasminogen activator inhibitor-1 (PAI-1), TNF-α, and leptin, can modulate airway epithelial and immune cell activation, thereby amplifying airway inflammation (57, 58). These mediators mainly drive non–type 2 inflammation, particularly neutrophilic inflammation. Since neutrophilic inflammation is widely recognised as poorly responsive to corticosteroids, this profile may be associated with corticosteroid insensitivity.

#### 4.3.2 Interleukin-17

Interleukin-17 (IL-17) is a key mediator of neutrophilic inflammation. In severe asthma, IL-17 has been implicated in corticosteroid insensitivity (59). In obesity, Th17 cells are increased, leading to elevated circulating IL-17 levels (60). Consistently, obese patients with asthma have shown increased expression of NLRP3 in sputum cells, along with higher IL-1β and IL-17 levels (61, 62).

IL-17 may promote corticosteroid insensitivity through several mechanisms. First, IL-17–driven neutrophilic inflammation creates a steroid-unresponsive inflammatory milieu. Second, IL-17 has been reported to directly induce GRβ expression in airway epithelial cells, thereby conferring corticosteroid insensitivity (63).

#### 4.3.3 Leptin

Leptin, an adipose-derived hormone that centrally regulates body weight, is elevated in peripheral blood in obesity due to reduced central leptin sensitivity. Peripheral leptin sensitivity, however, is preserved in tissues including immune cells (64). Consequently, elevated circulating leptin exerts multiple immunomodulatory effects in obese individuals. Leptin has been shown to activate the PI3K pathway (65). and enhance oxidative bursts in human monocytes (66). Although no clinical studies have directly demonstrated that obesity-related hyperleptinaemia induces corticosteroid insensitivity, both PI3K activation and oxidative stress are recognised contributors, suggesting that leptin may promote corticosteroid insensitivity through these mechanisms.

Leptin also has promoted Th17 cell activation (67, 68).leading to enhanced IL-17 signalling. Since IL-17 drives neutrophilic inflammation and induces GRβ expression in airway epithelial cells (64) leptin-mediated Th17/IL-17 activation may exacerbate corticosteroid insensitivity by combining inflammatory amplification with dysregulated GR expression.

## 5 Systemic modulators of corticosteroid resistance in obese asthma

### 5.1 Obstructive sleep apnoea syndrome

Obstructive sleep apnoea syndrome (OSAS) is characterised by recurrent upper airway obstruction during sleep, resulting in brief episodes of apnoea or hypopnoea. These events are accompanied by cycles of hypoxaemia and reoxygenation, persistent inspiratory effort against the occluded airway, and frequent sleep fragmentation. Obesity is a major risk factor for the development of OSAS. The prevalence of OSAS was reported to be markedly higher in obese individuals than in those of normal weight (69). Recent epidemiological data indicated that approximately 70% of obese adults (BMI ≥ 30 kg/m^2^) were diagnosed with OSAS (70). In OSAS, repetitive cycles of hypoxia and reoxygenation increase systemic oxidative stress (71, 72). This heightened oxidative stress is thought to contribute to corticosteroid insensitivity. Supporting this, *ex vivo* analyses of samples from patients with OSAS demonstrated associations between elevated oxidative stress, reduced HDAC2 expression, and corticosteroid resistance in patients with OSAS and asthma (73). Moreover, chronic intermittent hypoxia, a hallmark of OSAS, has been reported to activate the p38 MAPK signalling pathway, thereby promoting corticosteroid insensitivity (74). In addition, frequent sleep fragmentation disrupts the normal circadian rhythm of corticosterone, abolishing the typical early-morning peak and nocturnal trough, and significantly increasing circulating corticosterone levels (75). This alteration may downregulate GRs.

Taken together, these findings suggest that, in patients with OSAS, not only coexisting obesity but also OSAS-specific mechanisms promote corticosteroid resistance. Supporting this notion, an observational study reported a higher frequency of severe asthma exacerbations in patients with asthma and concomitant OSAS (76), likely reflecting the impact of these mechanisms.

### 5.2 Metabolic dysfunction–associated steatotic liver disease

Metabolic dysfunction–associated steatotic liver disease (MASLD), formerly referred to as non-alcoholic fatty liver disease (NAFLD), is characterised by excessive mitochondrial ROS production in hepatocytes, driven by lipid overload–induced β-oxidation and overactivation of the electron transport chain. This process is accompanied by insulin resistance and chronic inflammation (77). Systemic oxidative stress is also elevated in MASLD (77). Although no data are currently available on oxidative stress within the airways of patients with MASLD, systemic oxidative stress resulting from hepatocyte mitochondrial ROS overproduction may promote corticosteroid insensitivity.

### 5.3 Impaired antiviral immunity

Viral infections may contribute to reduced corticosteroid sensitivity. Respiratory syncytial virus (RSV), rhinovirus, and influenza virus infections have been shown to impair the anti-inflammatory effects of corticosteroids via induction of transforming growth factor-β (78). Another study demonstrated that rhinovirus type 16 activated NF-κB and c-Jun N-terminal kinase (JNK) signalling in airway epithelial cells, leading to corticosteroid insensitivity (79).

Obesity has been associated with altered immune responses to viral infections (80). For instance, influenza vaccine antibody titres declined significantly, and CD8^+^ T-cell responses were impaired in obese individuals compared with those of healthy weight (81). In a prospective study of 1,022 vaccinated adults, the risk of developing influenza was approximately twice as high in obese individuals as in non-obese individuals (82). Obesity-associated impairment of antiviral immunity may therefore increase susceptibility to recurrent viral infections, potentially contributing to corticosteroid insensitivity.

### 5.4 Vitamin D deficiency

Vitamin D has been implicated in the activity of various chronic diseases. In asthma, a study of 280 adults demonstrated that serum 25-hydroxyvitamin D concentrations were significantly related to both asthma severity and control (83). The prevalence of vitamin D deficiency was highest among patients with severe, poorly controlled asthma (83). Furthermore, a meta-analysis of clinical trials found that vitamin D supplementation reduced the incidence of asthma exacerbations (84). Regarding corticosteroid sensitivity, vitamin D concentrations have been directly associated with corticosteroid sensitivity, measured by dexamethasone-induced mitogen-activated protein kinase phosphatase 1 (MKP-1) inductions (85). Particularly in patients not using ICS, a dose-response relationship was observed, with MKP-1 induction increasing by 0.05-fold per 1 ng/mL rise in vitamin D levels (85).

Obesity has been linked to reduced serum vitamin D concentrations (86, 87) Additionally, obese individuals have shown poor responsiveness to vitamin D supplementation (86). Obesity-associated vitamin D insufficiency is likely due to reduced bioavailability from cutaneous and dietary sources as a result of sequestration in adipose tissue (86). Collectively, these findings suggest that obesity-related vitamin D deficiency may contribute to impaired corticosteroid sensitivity in obese patients with asthma.

### 5.5 Modification of corticosteroid pharmacokinetics

Altered corticosteroid pharmacokinetics have also been reported in obesity. In obese patients receiving oral prednisolone, reduced gastrointestinal absorption and increased systemic clearance of prednisolone were observed (88). When oral corticosteroid therapy is required, these pharmacokinetic changes may contribute to the apparent reduction in corticosteroid sensitivity.

## 6 Lifestyle factors contributing to corticosteroid resistance in obese asthma

### 6.1 Advanced glycation end products

Hyperglycaemia and obesity promote protein glycation, ultimately leading to the formation of advanced glycation end products (AGEs). AGEs possess both pro-inflammatory properties and the capacity to induce cell death (89, 90). A study demonstrated that human THP-1 macrophage-like cells stimulated with dietary AGEs exhibited reduced corticosteroid sensitivity compared with those stimulated with lipopolysaccharides. This finding suggests that AGE-induced inflammation contributes to corticosteroid insensitivity (91). It was further suggested that AGEs have increased intracellular ROS, which contributes to the development of corticosteroid insensitivity (91). Dietary patterns characteristic of obesity, such as high-fat and high-sugar intake, and obesity-related metabolic abnormalities, such as hyperglycaemia, and ageing are known to accelerate AGE accumulation. The increased AGE levels associated with obesity may therefore contribute to diminished corticosteroid responsiveness.

### 6.2 Physical inactivity

Several studies have demonstrated the beneficial effects of exercise on asthma control (92, 93). Adults with asthma who lacked regular exercise habits were enrolled in a controlled trial comparing supervised exercise (three sessions weekly) with standard care. Patients in the exercise group achieved a 24% reduction in maintenance inhaled corticosteroid dose regardless of baseline BMI, whilst the control group showed no reduction in ICS requirements (94). Experimental evidence has supported exercise-induced antioxidant responses; treadmill exercise in COPD models increased pulmonary Nrf2 expression, suggesting enhanced antioxidant pathway activation (95). A review of exercise effects on human PBMCs reported that appropriate exercise enhanced their antioxidant capacity (96).

Direct evidence linking exercise to improved corticosteroid sensitivity in human airways or immune cells remains limited. However, extensive research confirms exercise-mediated reductions in oxidative stress. This pathway likely underlies exercise-induced improvements in corticosteroid sensitivity. Physical inactivity, commonly observed in obese individuals, may therefore contribute to reduced corticosteroid responsiveness in this population.

## 7 Therapeutic perspectives to restore corticosteroid sensitivity in obese asthma

### 7.1 Lifestyle modification in obese individuals: weight reduction, dietary intervention, and exercise

Weight reduction is the most critical intervention in the treatment of obesity-related diseases, and obesity-related asthma is no exception. Numerous studies have demonstrated that weight loss leads to improved asthma control in obese asthma. Direct evidence also showed that weight reduction restored corticosteroid sensitivity: patients with obesity and asthma underwent bariatric surgery, and PBMCs were collected before and after surgery to assess corticosteroid sensitivity (97). A significant improvement in corticosteroid sensitivity in PBMCs was observed after surgery (97). This finding suggests that weight loss can restore obesity-related corticosteroid insensitivity. Therefore, weight reduction should be considered the primary therapeutic approach whenever feasible.

Dietary interventions not only contribute to weight reduction but may also directly improve corticosteroid sensitivity. Avoiding excessive carbohydrate intake can suppress the production of AGEs, thereby potentially preventing AGE-induced corticosteroid insensitivity (91). Exercise interventions have been reported to exert benefits beyond weight loss, directly improving asthma control. In patients with asthma, exercise was shown to reduce the required dose of ICS and to attenuate oxidative stress, irrespective of BMI (95, 96). Reduction in oxidative stress is expected to enhance corticosteroid sensitivity. Thus, exercise is recommended as an adjunctive therapy.

Patients with obesity-related asthma are also more likely to present with obesity-related comorbidities. These comorbidities contribute to both asthma severity and corticosteroid insensitivity, highlighting the importance of their appropriate management. In particular, the management of OSAS is of critical importance. Treatment of OSAS can reduce oxidative stress, potentially restoring corticosteroid sensitivity. Moreover, continuous positive airway pressure (CPAP) therapy for OSAS was shown to increase the GRα/GRβ ratio (98). GRα is essential for glucocorticoid action, whereas GRβ acts as a dominant-negative inhibitor of GRα-mediated transactivation. Thus, improvement in this ratio may also enhance corticosteroid sensitivity.

### 7.2 Pharmacological approaches

In the management of patients with asthma and obesity, standard asthma therapy, primarily ICS, should first be initiated in parallel with the non-pharmacological interventions described above. This approach achieves improved asthma control in a subset of patients. However, a substantial proportion of patients either fail to respond adequately to this treatment or are unable to achieve meaningful weight reduction. In such cases, treatment decisions should be guided by whether type 2 inflammation is present. When type 2 inflammatory features are identified, the use of biologic agents should be considered. Biologics have been reported to be effective in patients with obese asthma who meet established eligibility criteria (99–102). By contrast, pharmacological options for patients with a predominance of non–type 2 inflammation remain extremely limited, particularly those capable of restoring obesity-related corticosteroid insensitivity. The development of drugs specifically targeting this pathophysiology is therefore urgently needed.

#### 7.2.1 Suppression of oxidative stress

In individuals with obesity, systemic oxidative stress derived from adipose tissue contributes to the progression of various obesity-related diseases. In an obese mouse model, pharmacological blockade of oxidative stress within adipose tissue suppressed ectopic fat accumulation and ameliorated MASLD (103). A similar mechanism may underlie improvements in airway inflammation and corticosteroid resistance in obesity-related asthma. However, the development of antioxidant drugs for airway diseases characterised by increased oxidative stress—irrespective of obesity status—remains far from clinical application.

Within this context, drug repositioning potentially offers a promising strategy. To et al. demonstrated that PBMCs from asthma patients with a high body fat percentage exhibited reduced corticosteroid sensitivity compared with those from patients with a normal body fat percentage (13). When cultured in the presence of metformin, these PBMCs showed restored corticosteroid sensitivity accompanied by a reduction in oxidative stress markers in the supernatant (13). This effect was not observed in PBMCs from patients with normal body fat percentage. This finding suggests that metformin restores corticosteroid sensitivity by suppressing oxidative stress (104–107). Given that metformin is already widely used for the treatment of diabetes and has a well-established safety profile, it may represent a promising candidate for repurposing as a therapeutic agent for obesity-related asthma.

#### 7.2.2 Potential of phosphoinositide 3-kinase δ-specific inhibition

Oxidative stress plays a central role in the pathophysiology of obesity-associated asthma. In addition to directly suppressing oxidative stress, blocking downstream pathways through which oxidative stress impairs corticosteroid sensitivity represents a potential therapeutic strategy. Oxidative stress initially activates the PI3K–AKT pathway (32, 35), which in turn phosphorylates HDAC2, leading to reduced enzymatic activity and consequent impairment of corticosteroid sensitivity (36). Within this pathway, PI3Kδ has been identified as a key protein. In a smoke-induced inflammatory model, PI3Kδ inhibition restored corticosteroid function, providing evidence that the PI3Kδ pathway is pivotal in driving corticosteroid insensitivity (31). PI3Kδ-selective inhibitors are currently under development for other diseases, and their future application to obesity-associated asthma warrants consideration.

Among existing drugs, low-dose theophylline has attracted attention for its potential to restore corticosteroid sensitivity. Low-dose theophylline selectively inhibited oxidative stress–induced activation of PI3Kδ, thereby restoring HDAC2 activity and improving corticosteroid sensitivity (32, 108). This effect occurred at concentrations around 1 μM, which were too low to elicit phosphodiesterase inhibition or adenosine receptor antagonism. In humans, the optimal therapeutic range has been proposed to be at plasma concentrations below 5–10 mg/L (109), where neither bronchodilator nor adenosine antagonistic effects occur. Thus, low-dose theophylline may offer a promising and safe therapeutic option for improving corticosteroid sensitivity in obesity-associated asthma.

#### 7.2.3 Potential of leptin antagonism

Hyperleptinaemia may contribute to reduced corticosteroid sensitivity (65–68). Consequently, controlling elevated leptin levels could potentially improve corticosteroid sensitivity. Research on the control of hyperleptinaemia has been conducted primarily in the context of therapies for obesity-related disorders. These studies focus on two main strategies: enhancing central leptin sensitivity and antagonising peripheral leptin activity (110). However, these approaches remain at an early investigational stage and are far from clinical application.

### 7.3 Metformin as a potential therapeutic agent

Metformin is widely used as an antidiabetic agent and is known to exert diverse pharmacological effects beyond glycaemic control (111). Several studies have suggested a potential benefit of metformin in asthma management. In a retrospective study of adolescents and young adults aged 12–21 years with asthma, those receiving metformin experienced fewer exacerbations compared with BMI-matched individuals not receiving the drug (112). Similarly, in patients with asthma and comorbid diabetes, metformin use was associated with a reduced risk of asthma exacerbations (113–115).

Although these clinical studies did not specifically evaluate the relationship between metformin and corticosteroid sensitivity, its known ability to activate AMPK and attenuate oxidative stress suggests a potential role in restoring obesity-associated reductions in corticosteroid sensitivity. Supporting this hypothesis, an ex vivo study demonstrated that metformin restored corticosteroid sensitivity (13). Clinical trials are warranted to investigate whether metformin can improve corticosteroid sensitivity in patients with obesity-related asthma.

### 7.4 Other considerations

Serum vitamin D levels are frequently reduced in individuals with obesity (86, 87). Vitamin D deficiency has also been linked to asthma exacerbations and reduced corticosteroid sensitivity. Therefore, in obese patients, it may be important to assess serum vitamin D levels and consider supplementation when appropriate.

Glucagon-like peptide-1 (GLP-1) receptor agonists, which are approved for the treatment of diabetes and obesity, exert pleiotropic effects across various organs beyond their metabolic actions (116). GLP-1 has been reported to modulate advanced glycation end product (AGE)–receptor for AGE (RAGE) interactions (117), thereby suppressing inflammation. Characteristic dietary patterns in obesity increase AGE accumulation, and excess AGEs have been implicated in impaired corticosteroid sensitivity through mechanisms involving oxidative stress. Consequently, GLP-1 receptor agonists may improve corticosteroid sensitivity by inhibiting AGE–RAGE interactions and blocking downstream AGE-related signalling pathways.

## 8 Conclusion and future directions

**Figure 1:**
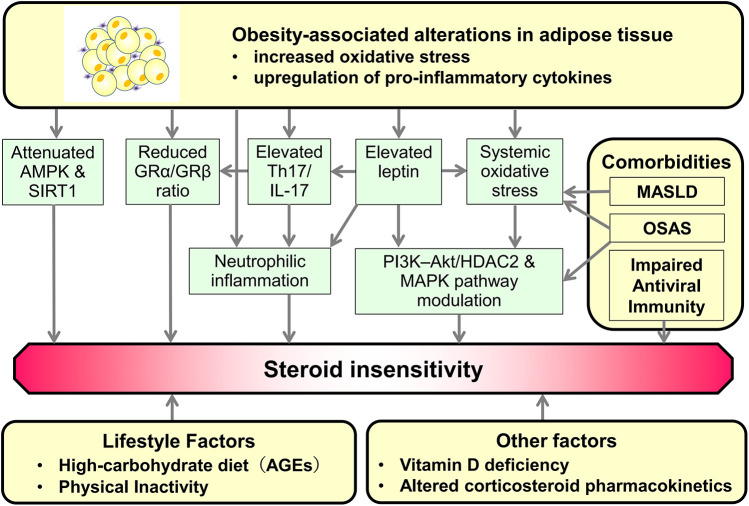
Potential mechanisms underlying the association between obesity-related factors and steroid insensitivity in obese asthma. Adipose tissue hypertrophy in obesity is characterised by increased oxidative stress and the upregulation of pro-inflammatory cytokines. These changes induce attenuated AMPK and SIRT1 expression and activity, a reduced GRα/GRβ ratio, elevated Th17/IL-17 responses, increased leptin secretion, and enhanced systemic oxidative stress. These alterations interact to promote neutrophilic inflammation and to modify multiple intracellular signalling pathways, ultimately leading to the development of steroid insensitivity. In addition, obesity-related comorbidities, lifestyle factors, and other systemic conditions further exacerbate the establishment of steroid-insensitive asthma. AMPK, adenosine monophosphate-activated protein kinase; SIRT1, sirtuin 1; GR, glucocorticoid receptor; Th17, T helper 17; IL-17, interleukin-17; PI3K, phosphoinositide 3-kinase; MAPK, mitogen-activated protein kinase; HDAC2, histone deacetylase 2; OSAS, obstructive sleep apnoea syndrome; MASLD, metabolic dysfunction–associated steatotic liver disease; AGEs, advanced glycation end-products.

Obesity-associated asthma represents a complex clinical entity characterised by impaired corticosteroid sensitivity, increased risk of exacerbations, and poorer disease control. Among these features, reduced corticosteroid sensitivity plays a pivotal role in worsening the disease. Corticosteroid insensitivity leads to more frequent exacerbations, often necessitating systemic corticosteroid use. This treatment, in turn, promotes further weight gain and metabolic dysfunction, perpetuating a vicious cycle of poor asthma control and progressive obesity. Multiple obesity-related factors contribute to this pathophysiology. Increasing evidence implicates adipose tissue-derived adipocytokines, oxidative stress, dysregulated immune responses, and obesity-associated systemic comorbidities and lifestyle factors in the development of corticosteroid insensitivity (Figure 1).

Therapeutic strategies must therefore extend beyond standard asthma pharmacotherapy. Lifestyle interventions, including weight loss, structured exercise, and dietary modification, are central to management and can improve both asthma control and corticosteroid responsiveness (Table 1). However, their effects are often limited, highlighting the urgent need for targeted pharmacological approaches. Potential avenues include agents that suppress oxidative stress or selective PI3Kδ inhibitors, although these remain distant from clinical application. Drug-repurposing candidates such as low-dose theophylline, metformin, and GLP-1 receptor agonists are of particular interest given their well-established safety profiles and mechanistic potential. These agents may represent promising options for translation into clinical practice through drug repurposing in the management of obesity-related asthma (Table 1).

**Table 1:** Therapeutic options for restoring corticosteroid sensitivity in obese asthma.

Therapeutic approach	Primary mechanism(s) targeted	Effect on corticosteroid sensitivity	Evidence level	Clinical applicability
Lifestyle modifications				
Weight reduction (including bariatric surgery)	• Reduces systemic inflammation • Decreases oxidative stress from adipose tissue • Reduces leptin levels	Direct restoration of corticosteroid sensitivity in PBMCs demonstrated post-bariatric surgery (97)	Clinical evidence	First-line intervention; feasible when possible
Dietary intervention (carbohydrate restriction)	• Suppresses AGE production • Reduces AGE-induced oxidative stress	Prevents AGE-induced corticosteroid insensitivity (91)	Mechanistic evidence	Adjunctive therapy; contributes to weight loss
Exercise	• Reduces oxidative stress • Improves metabolic parameters	Attenuates oxidative stress, reduces ICS dose requirement (94–96)	Clinical evidence	Recommended adjunctive therapy; benefits independent of weight loss
Comorbidity management				
Obstructive sleep apnoea syndrome	• Reduces oxidative stress • Increases GRα/GRβ ratio (98)	Enhances corticosteroid sensitivity through improved GR signalling and reduced oxidative stress	Clinical evidence	Critical in obese asthma patients with OSAS
Standard therapy				
Inhaled corticosteroids	• Suppresses airway inflammation	Variable response; may be ineffective in obesity-related corticosteroid resistance	Standard practice	First-line pharmacotherapy with lifestyle interventions
Biologic agents	• Targets type 2 inflammation pathways	Effective in obese asthma with type 2 inflammation (99–102)	Clinical evidence	For type 2 inflammation-predominant obese asthma
Targeting oxidative stress				
Metformin	• Activates AMPK • Suppresses oxidative stress (104–107) • May modulate AGE-RAGE interactions	Restores corticosteroid sensitivity in PBMCs from high body fat percentage patients (13)	Ex vivo evidence	Promising repurposing candidate; established safety profile; clinical trials warranted
		Restores corticosteroid sensitivity by activation of AMPK (54)		
Targeting PI3K-AKT-HDAC2 pathway				
PI3Kδ-specific inhibitors	• Blocks PI3Kδ activation • Prevents HDAC2 phosphorylation • Restores HDAC2 activity	Restores corticosteroid function in smoke-induced inflammation model (31)	Preclinical evidence	Under development; future application warranted
Low-dose theophylline	• Selectively inhibits oxidative stress-induced PI3Kδ activation • Restores HDAC2 activity (32, 108)	Improves corticosteroid sensitivity at plasma concentrations <5–10 mg/L (109)	Mechanistic evidence	Promising and safe option; requires clinical validation in obese asthma
Targeting leptin				
Leptin antagonism	• Reduces hyperleptinemia effects (65–68)	Potential improvement in corticosteroid sensitivity	Early investigational	Far from clinical application
Other considerations				
Vitamin D supplementation	• Corrects vitamin D deficiency (86, 87) • Reduces inflammation	May improve corticosteroid sensitivity and reduce exacerbations	Clinical evidence	Consider assessment and supplementation in obese patients
GLP-1 receptor agonists	• Modulates AGE-RAGE interactions (117) • Suppresses AGE-related inflammation and oxidative stress • Promotes weight loss	May improve corticosteroid sensitivity through AGE pathway inhibition	Mechanistic hypothesis	Approved for diabetes/obesity; potential benefit in obese asthma

AGE, advanced glycation end product; AMPK, adenosine monophosphate-activated protein kinase; GLP-1, Glucagon-like peptide-1; GR, glucocorticoid receptor; HDAC2, histone deacetylase 2; ICS, inhaled corticosteroids; OSAS, obstructive sleep apnoea syndrome; PBMCs, peripheral blood mononuclear cells; PI3 K, phosphoinositide 3-kinase; RAGE, receptor for AGE.

Future research should prioritise elucidating the causal pathways underlying corticosteroid insensitivity through mechanistic studies, thereby guiding the rational development of targeted therapies. Given the availability of several drug-repositioning candidates, early clinical investigations of these agents are strongly warranted to accelerate progress towards effective therapies for this challenging phenotype.
